# Developmental pathways of depressive symptoms via parenting, self-evaluation and peer relationships in young people from 3 to 17 years old: evidence from ALSPAC

**DOI:** 10.1007/s00127-022-02416-6

**Published:** 2023-01-28

**Authors:** Mengya Zhao, Tamsin Ford, Margarita Panayiotou, Anke Karl

**Affiliations:** 1 University of Exeter, Exeter, UK; 2 University of Cambridge, Cambridge, UK; 3 The University of Manchester, Manchester, UK

**Keywords:** Parenting, Self-evaluation, Peer relationships, Depressive symptoms, Developmental pathway

## Abstract

**Purpose:**

Self-evaluation and interpersonal factors are theoretically and empirically linked to depression in young people. An improved understanding of the multifactorial developmental pathways that explain how these factors predict depression could inform intervention strategies.

**Methods:**

Using structural equation modeling, this study explored whether self-evaluation and interpersonal factors were associated with adolescent depressive symptoms in a population-based sample (*n* = 11,921; Avon Longitudinal Study of Parents and Children, ALSPAC), across four development stages: early and late childhood plus early and middle adolescence from 3 to 17 years old.

**Results:**

Early good parenting practices predicted self-esteem, fewer peer difficulties, good friendships and fewer depressive symptoms in late childhood development outcomes. Higher self-esteem and less negative self-concept mediated the effect of early good parenting practice on reduced depressive symptoms in middle adolescence. The hypothesized erosion pathway from depressive symptoms in late childhood via higher levels of negative self-concept in early adolescence to depressive symptoms in middle adolescence was also confirmed. Additionally, peer difficulties played a mediation role in developing depressive symptoms. Contrary to the hypothesis, poor friendships predicted fewer depressive symptoms. The analysis supported a developmental pathway in which good parenting practices in early childhood led to fewer peer difficulties in late childhood and to less negative self-concept in early adolescence, which in turn predicted fewer depressive symptoms in middle adolescence.

**Conclusion:**

The social-developmental origin of youth depressive symptoms was supported via the effect of peer relationships in late childhood on self-evaluation in early adolescence.

**Supplementary Information:**

The online version contains supplementary material available at 10.1007/s00127-022-02416-6.

## Introduction

Depression in adolescence affects employment, relational function and mental health in adulthood [14, 45]. Thus, it is critical to understand the developmental pathways of adolescent depressive symptoms. Self-evaluation as indicated by self-esteem [61] and interpersonal factors such as the style of parenting in childhood [13] and friendships [58] have been linked to depressive symptoms, but there is limited research examining the potential developmental pathways of adolescent depressive symptoms involving all these factors. Understanding such developmental pathways could identify targets for intervention and prevention of depression. Therefore, the current study aims to explore the developmental pathways across different developmental periods.

Negative self-evaluation is a major risk factor for depressive symptoms [3, 37, 50]. Self-evaluation is defined as the way individuals appraise themselves, including worth and attributes [6]. It comprises self-esteem, referring to one’s evaluation of worth [53] and self-concept, referring to how young people see themselves [43]. The association between self-evaluation and depressive symptoms has been explained by two opposite theoretical perspectives, the vulnerability model and the scar model (see [61]). Whereas the vulnerability model states that negative self-evaluation is the cause of depression, the scar model postulates that depression leads to negative self-evaluation. Although empirical studies have supported both models, the evidence for the vulnerability model is currently stronger [52]. Given the importance of self-evaluation for depression, it is critical to understand the developmental pathways of depressive symptoms in relation to self-evaluation.

The development of self-evaluation cannot be understood independent of interpersonal factors, such as parenting and peer relationships, which are closely intertwined [1, 26–28, 33]. However, there is limited research exploring the longitudinal association between self-evaluation and depressive symptoms from an interpersonal perspective. Good parenting practices in early life is considered the origin of self-evaluation via the development of Internal Working Models (IWMs) about the self and others as postulated in attachment theory [1]. Children whose parents were warm and sensitive to their needs in early life are more likely to develop a positive self-evaluation, for example, that they are loveable. Parental warmth predicted self-esteem in Mexican-origin youth from age 10 to 16 [33]. A recent systematic review [13] highlighted that the evidence for the association between positive childhood parenting and adolescent depression relies heavily on cross-sectional research or longitudinal studies that covered only a limited developmental period. Only two longitudinal studies supported a significant association between positive parenting in childhood and depression in adolescence [19, 66].

Parenting practices are also hypothesized as a predictor of positive peer relationships via the same IWMs of self and others [1], as posited by the Interpersonal Model of Youth Depression [55]. The latter is a developmental pathway from early family disruption via relationship disruption to depression [55]. Although the association between parenting and peer relationships was supported by several studies [17, 38, 40], most investigated parenting in late childhood or adolescence rather than focusing on early life parenting. Thus, the evidence supporting the assumptions of attachment theory is scarce.

Peer relationships may also play a role in forming self-esteem [26] and depressive symptoms [55]. Peer relationships include on one hand peer difficulties (defined as having problems getting along with peers) and on the other hand friendships (defined as feeling support from friends, comfortable talking problems with friends and happy with friendships). For peer relationships and youth depression, three different longitudinal associations have been hypothesized with empirical support for each. The interpersonal risk model posits that poor interpersonal relationships cause youth depression [32], and indeed conflicts with friends were found to predict depressive symptoms [68]. The symptoms-driven model states that youth depression erodes interpersonal relationships [32], and depressive symptoms predict peer rejections [34, 68]. The transactional model suggests that depression and interpersonal relationships influence each other [32] as was supported by bidirectional links between depressive symptoms and peer victimization, peer acceptance and support by friends [20, 54, 68].

There was also no consistent evidence supporting the nature of the associations between self-evaluation, interpersonal relationships and depressive symptoms. This is due to reciprocal associations between self-evaluation and interpersonal relationships [26, 64] but also inconsistencies in the mediation of depressive symptoms. On one hand, self-esteem mediated the association between peer victimization and depression in a one-year longitudinal study [49]. In contrast, an indirect pathway from self-evaluation to depressive symptoms via peer relationships was also found [51], which suggested that negative self-evaluation may cause maladaptive social interactions and poor interpersonal relationships, which subsequently lead to depression. Yet another study found an indirect pathway from self-esteem to peer victimization via depressive symptoms in adolescents from 12 to 17 years old [56].

Taken together, theory and empirical evidence suggests cross-sectional and longitudinal associations between self-evaluation, interpersonal factors and depressive symptoms. However, because self-evaluation and peer relationships are reciprocally associated with each other, it is as yet unclear whether there is a developmental pathway from childhood experience of parenting to adolescent depression involving self-evaluation and peer relationships. Partial support comes from a 6-year longitudinal study in which self-esteem was a mediator from dysfunctional parenting to peer attachment with three-time points [38]. However, Lim [38] did not assess depression. There is also evidence for a different developmental trajectory between self-evaluation and peer relationships, in which poor peer relationships are the origin of negative self-evaluation [15], and is concordant with the social-developmental origin of negative self-evaluation of depressive symptoms in adolescence [7]

**Fig. 1:**
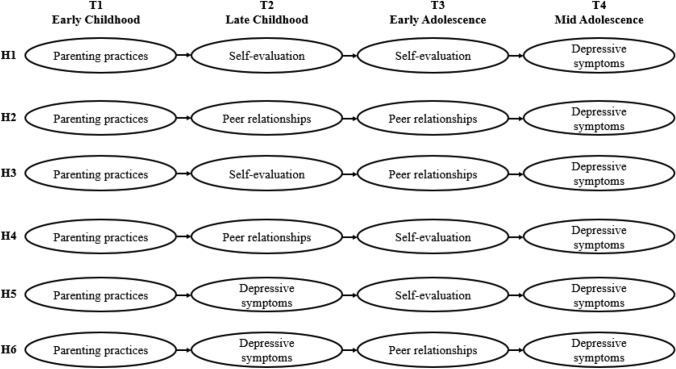
The hypothesized mediation pathway model. (1) We also predicted that parenting practice would have direct associations with the development outcomes in early adolescence and middle adolescence. Also, self-esteem would have direct associations with the development outcomes in middle adolescence. (2) In the current study, peer relationships indicated by peer difficulties and friendships; Self-evaluation at T2 means self-esteem but at T3 means self-concept

Given the uncertain developmental pathway between self-evaluation, peer relationships, and depressive symptoms, it is important to assess self-evaluation, peer relationships and depressive symptoms at several different time points through childhood and adolescence to investigate the IWMs and address the current gaps (e.g., [13]. We therefore aimed to explore the developmental pathways of depressive symptoms across four developmental stages using the Avon Longitudinal Study of Parents and Children (ALSPAC) database (Fig. 1). ALSPAC is a population-based database involving children born between 1991 and 1992 in Avon, England. Specifically, we included parenting practices in early childhood, self-evaluation in late childhood and early adolescence, peer relationships and depressive symptoms in late childhood and early and middle adolescence.

Figure 1 illustrates our hypothesized pathways; in addition to two pathways, (H1) via self-evaluation based on the vulnerability model and (H2) via peer relationships based on interpersonal risk model, the current study proposed several additional pathways from parenting. Specifically, potential risk factors contribute to depressive symptoms: (H3) via negative self-evaluation (i.e., self-esteem) in late childhood to poor peer relationships in early adolescence and (H4) via poor peer relationships in late childhood and then negative self-evaluation (i.e., self-concept) to test social-developmental origin of self-evaluation assumption [7]. Two further pathways were hypothesized suggesting that depression erodes functions in other domains: (H5) via depressive symptoms in late childhood leading to negative self-evaluation to investigate the scar model, (H6) via depressive symptoms in late childhood and subsequent poor peer relationships to investigate symptoms-driven model.

## Methods

### Participants

Data were from ALSPAC (http://www.alspac.bris.ac.uk). All pregnant women whose estimated delivery date fell between 1 April 1991 and 31 December 1992 and living in one of the three health administration districts Southmead NHS District Health Authorities (DHA), Frenchay DHA and Bristol and Weston DHA [5] were invited. In total, 15,247 eligible pregnant women enrolled in ALSPAC (detailed recruitment flow diagram please see [5], and 15,645 children were in the database (children’s sex at birth: 49.2% boys, 37% girls, and 3.9% children having missing information regarding sex, cohort profile please see Fraser et al. [21]. In this study, 11,921 children were included because there were 3724 cases missing required variables. Ethical approval was obtained from the ALSPAC Law and Ethics Committee and the CLES Ethics Committees of the University (eCLESPsy001234 v2.1). We lacked access to demographic variables (e.g., ethnicity, SES), so we cannot present sample characteristics and compare participants included in the model with those not included due to missingness, however, the ALSPAC sample has been widely characterised elsewhere as broadly representative from the sample from which it was recruited.

### Measures

A detailed justification of the measures and procedures to establish its psychometric properties using confirmatory factor analysis (CFA) can be found in the supplementary material. Roughly, there were four-time waves across four developmental stages (see Table 1): T1 indicated early childhood (i.e., 3 years 2 months); T2 indicated late childhood (i.e., 9.5–10.5 years); T3 indicated early adolescence (i.e., 13 years to 13 years 10 months); T4 indicated middle adolescence (i.e., 16.5–17.5 years).

**Table 1:** Correlation, mean, standard deviation, skewness table

	SE	SC	PD_T1	PD_T2	PD_T3	PF_T1	PF_T2	PF_T3	DS_T1	DS_T2	DS_T3	Mean	Standard deviation	Skewness	Range	Sample size
PP	0.10	− 0.07	− 0.10	− 0.08	− 0.06	− 0.06	− 0.07	**< 0.01**	− 0.05	**< 0.01**	− **0.02**	25.11	3.14	− 1.01	2–30	10,080
SE	–	− 0.29	− 0.26	− 0.20	− 0.18	− 0.23	− 0.20	− 0.16	− 0.30	− 0.19	− 0.19	30.89	3.95	− 1.43	7–35	7336
NSC		–	0.27	0.32	0.31	0.16	0.40	0.30	0.26	0.55	0.38	21.53	3.96	0.03	9–39	6606
PD_T1			–	0.70	0.53	0.30	0.24	0.21	0.33	0.19	0.15	1.08	1.45	1.81	0–10	7413
PD_T2				–	0.67	0.34	0.32	0.23	0.31	0.20	0.18	1.15	1.57	1.86	0–10	6518
PD_T3					–	0.24	0.29	0.29	0.24	0.19	0.23	1.02	1.39	1.92	0–19	5064
PF_T1						–	0.40	0.22	0.36	0.09	0.08	1.08	1.30	1.56	0–8	7294
PF_T2							–	0.34	0.25	0.27	0.12	.85	1.16	1.63	0–8	6081
PF_T3								–	0.14	0.17	0.23	1.53	1.58	1.13	0–9	4042
DS_T1									–	0.31	0.27	4.04	3.51	1.42	0–23	7362
DS_T2										–	0.43	4.92	4.49	1.46	0–26	6017
DS_T3											–	5.91	5.64	1.37	0–26	4995

Correlations are standardised. The descriptive analysis results are based on the sum score of the main variables, and the sample size is the number of completed cases; Range is the range of the minimum value and maximum value. Numbers in bold means non-significance; ps ≤ 0.001

PP assessed at 3 years 2 months; SE assessed at 9 years 7 months; SC assessed as 13 years 10 months; PD_T1 assessed at 9 years 7 months; PD_T2; 13 years 1 month; PD_T3 assessed at 16 years 6 months; PF_T1 assessed at 10 years 6 months; PF_T2 assessed at 13 years 6 months; PF_T3 assessed at 17 years 6 months; DS_1 assessed at 10 years 6 months; DS_2 assessed at 13 years 6 months; DS_3 assessed at 16 years 6 months

*PP* parenting practice, *SE* self-esteem, *NSC* negative self-concept, *PD* peer difficulties, *PF* poor friendships, *DS* depressive symptoms

#### Mother-reported parenting practice

Self-reported maternal parenting score at T1 was provided by the ALSPAC team, which was calculated based on the sum of the frequency of ten parenting activities with children, including, bathing, feeding, singing, playing with toys etc., on a 4-point Likert scale (from “often” 3 to “never” 0); the total score ranged from 0 to 30, and higher score means good parenting practice.

#### Self-evaluation

Self-evaluation was assessed by self-esteem at T2 and self-concept at T3. For self-esteem, seven items were selected from the Self-Description Questionnaire [41]. Children responded to a five-point Likert scale from 1 “not true” to 5 “true” to rate items (e.g., “In general, I like the way I am”). Scores ranged from 5 to 35, and higher scores indicated higher levels of self-esteem. For self-concept, nine items were selected for this study from Self-Image Profile [8] based on words previously used in a self-referential task for the study of self-compassion developed by Kirschner [31]. Children responded to a 5-point Likert scale from 5 (always) to 1 (never) to rate a list of words to describe themselves (e.g., “kind”) at T3. Scores ranged from 8 to 40, and higher scores indicated negative self-concept. The internal consistency for self-esteem and self-concept were 0.77 and 0.66, respectively.

#### Peer relationships

There are two variables assessing peer relationships at T2, T3 and T4; child-reported friendships and mother-reported peer difficulties. For friendships, three items (e.g., “talk about problems”) from the Cambridge Hormones and Moods project Friendship questionnaire [24] were rated on a 4-point scale from 1 (most of the time) to 4 (not at all). Higher scores indicated poor friendships. Peer difficulties were assessed by a 5-item peer difficulties subscale (e.g., “picked on or bullied by other children”) from the parent-report Strengths and Difficulties Questionnaire [22] on a 3-point scale from 0 (not true) to 2 (true). Scores ranged from 0 to 10 and higher scores indicated more peer difficulties. Internal consistency was *α* = 0.63/0.65/0.58 for peer difficulties, and *α* = 0.48/0.44/0.71 for friendships T2/T3/T4.

#### Depressive symptoms

Child-reported depressive symptoms were assessed by the 13-item short Mood and Feelings Questionnaire (SMFQ) [2] at T2, T3 and T4. SMFQ assessed the frequency of depressive symptoms (e.g., “I felt lonely”) in the past 2 weeks on a 3-point scale from 0 (“not true”) to 2 (“true”). The score of SMFQ ranged from 0 to 26, and higher scores indicated high levels of depressive symptoms. Internal consistency was *α* = 0.80/0.87/0.91, T2/T3/T4.

### Statistical analyses

SPSS 25.0 was used for internal consistency. MPlus 8.4 was used for structural equation modeling (SEM). There was substantial attrition in ALSPAC for children from 3 to 17 years. Therefore, the full completed case analysis (FCC, *n* = 1292) is more likely to suffer from selection bias because the pattern of missing data is not missing completely at random (Table S12). Although data reported via Likert-type scales should be treated as ordinal data for which the weighted least squares mean and variance adjusted estimation (WLSMV) in Mplus was recommended [48], robust maximum likelihood (MLR) estimation and full information maximum likelihood (FIML) were used for data analysis to allow missing data management for the primary analyses [12] whilst accounting for the non-normal distribution [39].

The following data analyses were conducted. First, confirmatory factor analysis (CFA) was used to confirm the measurement constructs (see supplementary materials). We established one-factor structures for self-esteem, peer difficulties, friendships and depressive symptoms and a bifactor model for self-concept.

Second, a series of nested models were computed using longitudinal CFA to establish the measurement invariance (MI) across measurement waves [65]. We established partial scalar MI of peer difficulties and depressive symptoms, and we did not establish measurement invariance of friendships (see supplementary materials). To understand the potential impact of partial MI, we compared the main findings with the analysis in which we robustly constrained the factor loading and intercepts as recommended by Chen [11]. These comparisons revealed that our findings are stable.

Next, SEM with latent factors was used to explore the longitudinal associations between parenting, self, interpersonal relationships and depressive symptoms. Mediation analysis explored the indirect effects of self-evaluation and peer relationships on depressive symptoms in mid-adolescence.

We evaluated the model fit for CFA and SEM based on a joint consideration of the value of chi-square/degree of freedom (χ^2^/df, ≤ 5), the values of root mean square error of approximation (RMSEA, ≤ 0.08), standardized root mean square residual (SRMR, ≤ 0.06), comparative fit index (CFI, ≥ 0.90) and Tucker-Lewis index (TLI, ≥ 0.90) following standard recommendations [29, 42].

## Results

### Results of structural equation modeling

**Fig. 2:**
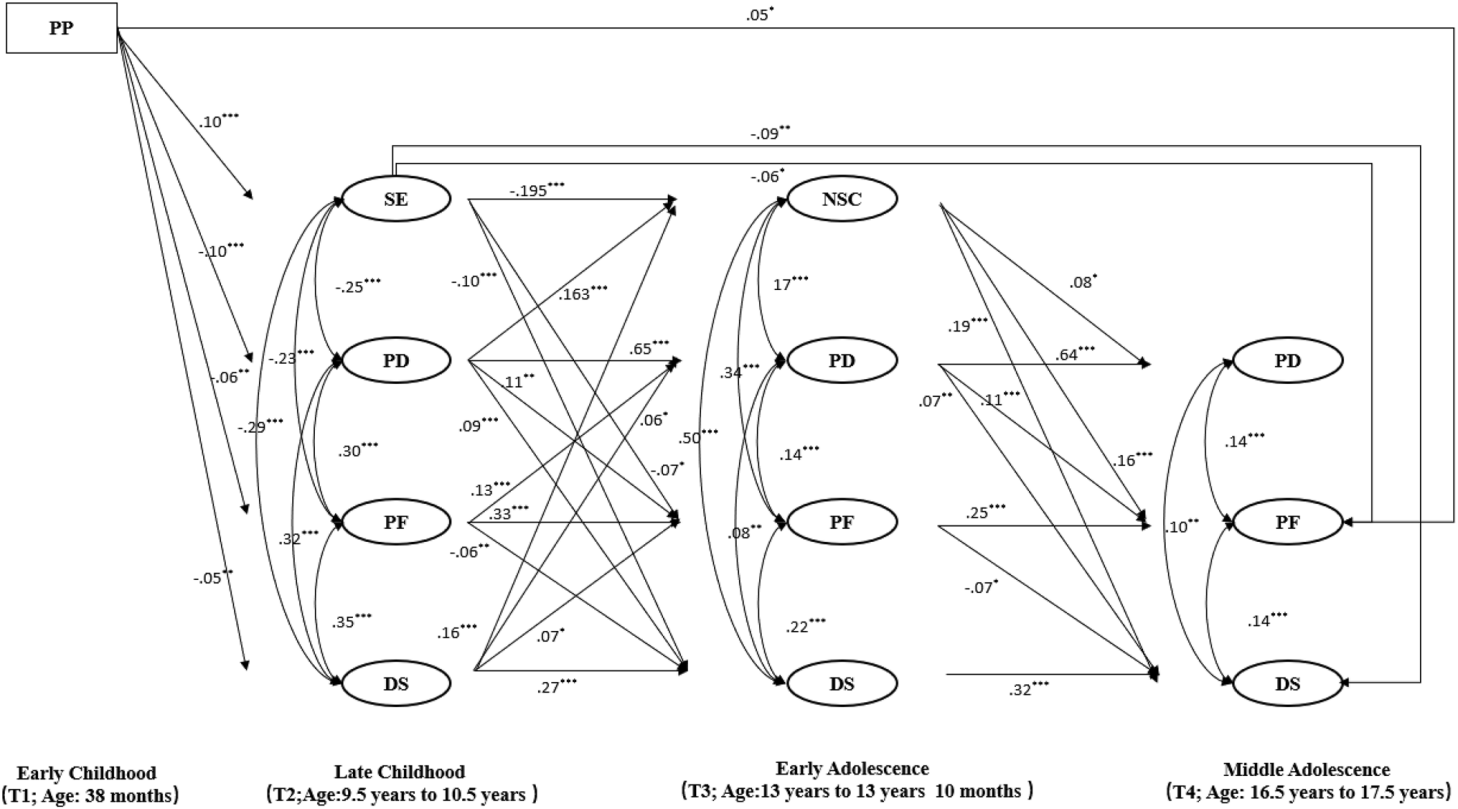
The pathway model: beta values are standardised. *N* = 11,921. *PP* parenting practice, *SE* self-esteem, *NSC* negative self-concept, *PD* peer difficulties, *PF* poor friendships, *DS* depressive symptoms, *p < 0.05; **p < 0.01; ***p < 0.001;

Descriptive statistics of variables see Table 1. The model fit of the pathway model (Fig. 2) was acceptable: *χ*^2^ (3013) = 13,160.32, *p* < 0.001, CFI = 0.909, TLI = 0.905, RMSEA = 0.017, SRMR = 0.035.

For the cross-sectional associations, as illustrated in Fig. 2, we found that low self-esteem was associated with poor friendships, peer difficulties and depressive symptoms at T2. Similarly, at T3, negative self-concept was positively associated with poor friendships, peer difficulties and depressive symptoms. As for interpersonal relationships, peer difficulties were positively correlated with depressive symptoms, and poor friendships were positively correlated with depressive symptoms at T2, T3 and T4. Also, peer difficulties were positively linked with poor friendships at T2, T3 and T4.

As for the stability of variables, mother-reported peer difficulties showed moderate stability across time points [*β* = 0.65/0.64, 95% CI (0.61 0.69)/95% CI (0.59, 0.69), T2 to T3/T3 to T4], and child-reported friendships showed moderate stability across time points [*β* = 0.33/0.25, 95% CI (0.26, 40)/95% CI (0.17, 0.33), T2 to T3/T3 to T4]. Similarly, depressive symptoms were moderately stable across time points [β = 0.27/0.32, 95% CI (0.23, 0.31)/95% CI (0.26, 37), T2 to T3/T3 to T4].

Higher parenting scores at T1 predicting higher self-esteem, fewer peer difficulties, better friendships and fewer depressive symptoms at T2, but oddly, poor friendships at T4 (|*β*|s <  = 0.10). T2 self-esteem predicted fewer depressive symptoms at T3 and T3 self-concept predicted T4 depressive symptoms. Also, self-esteem predicted T4 depressive symptoms. However, contrary to the hypothesis, poor friendships at T2 and T3 predicted fewer depressive symptoms T3 and T4, respectively. Peer difficulties predicted depressive symptoms at T3 and T4, but the longitudinal association between interpersonal factors and depressive symptoms were very small (|*β*|s < 0.10). Notably, T2 depressive symptoms predicted negative self-concept and poor friendships and more peer difficulties at T3, while T3 depressive symptoms did not significantly predict any variables at T4.

As for the longitudinal association between self and interpersonal relationships, self-esteem and self-concept positively predicted friendships at T3 and T4 respectively, but the effect on peer difficulties was only demonstrated for self-concept. Also, self-esteem directly influenced T4 friendships. T2 peer difficulties predicted negative self-concept but friendships did not predict self-concept.

### Mediation analysis of indirect pathways

Possible indirect pathways to T4 depressive symptoms were identified and statistically assessed. Several pathways were statistically significant (Table 2). The direct effect from T1 parenting to T4 depressive symptoms was not significant, but seven indirect pathways were supported. We also explored the pathways from T2 variables to T4 depressive symptoms. Two indirect pathways from T2 self-esteem were supported. Three indirect pathways from T2 friendships and three indirect pathways from T2 peer difficulties were supported. In contrast, only two indirect pathways from T2 depressive symptoms were supported.

**Table 2:** Indirect pathways of depressive symptoms at T4

Indirect paths		Standardised coefficients		95% CI	
Predictor	Mediator(s)	*β*	SE	LL	UL
From parenting practice (T1) to depressive symptoms(T4)					
PP(T1) →	SE(T2) →	− 0.008**	0.002	− 0.013	− 0.003
PP(T1) →	SE(T2) → NSC(T3) →	− 0.003***	0.001	− 0.005	− 0.002
PP(T1) →	PD(T2) → NSC(T3) →	− 0.003^**^	0.001	− 0.005	− 0.001
PP(T1) →	PD(T2) → PD(T3) →	− 0.005**	0.002	− 0.008	− 0.001
PP(T1) →	SE(T2) → DS(T3) →	− 0.003***	0.001	− 0.005	− 0.001
PP(T1) →	PD(T2) → DS(T3) →	− 0.003**	0.001	− 0.004	− 0.001
PP(T1) →	DS(T2) → DS(T3) →	− 0.004**	0.015	− 0.006	− 0.001
Effects from self-esteem(T2) to depressive symptoms (T4)					
SE(T2) →	NSC(T3) →	− 0.036***	0.008	− 0.052	− 0.020
SE(T2) →	DS(T3) →	− 0.032***	0.007	− 0.045	− 0.019
Effects from friendships(T2) to depressive symptoms (T4)					
FQ(T2) →	FQ(T3) →	− 0.023*	0.010	− 0.044	− 0.003
FQ(T2) →	PD(T3) →	0.010**	0.004	0.003	0.017
FQ(T2) →	DS(T3) →	− 0.018**	0.007	− 0.032	− 0.005
Effects from peer difficulties (T2) to depressive symptoms (T4)					
PD(T2) →	NSC(T3) →	0.030***	0.008	0.015	0.046
PD(T2) →	PD(T3) →	0.048**	0.015	0.019	0.078
PD(T2) →	DS(T3) →	0.028***	0.008	0.014	0.043
Effects from depressive symptoms (T2) to depressive symptoms (T4)					
DS(T2)	NSC(T3)	0.030**	0.009	0.011	0.048
DS(T2)	DS(T3)	0.085***	0.011	< 0.001	0.009

*N* = 11,921. PP indicated parenting practice; SE indicated self-esteem; NSC indicated negative self-concept; PD indicated peer difficulties; FQ indicated poor friendships; DS indicated depressive symptoms

*p < 0.05; **p < 0.01; ***p < 0.001

## Discussion

We explored the potential psychosocial developmental pathways to depressive symptoms, including early parenting, self-evaluation and peer relationships across childhood to middle adolescence. Our findings supported several pathways from parenting to depressive symptoms (H1, H2 and H4) and partially supported the scar model (H5). There was no evidence for H3 and H6. Contrary to our hypothesis, poor friendships predicted fewer depressive symptoms. Additionally, we found that *negative* developmental outcomes (e.g., low self-esteem, and peer difficulties) in late childhood increased depressive symptoms in middle adolescence via depressive symptoms in early adolescence.

Our findings support IWMs based on attachment theory [1, 63],suggesting that good parenting practices in early childhood predicted higher self-esteem, more positive peer relationships and lower depressive symptoms in late childhood. Our findings suggest that early responsive and warm parenting practices may have an important role in facilitating children’s positive and healthy self-evaluation, promoting good quality of peer relationships, and preventing depressive symptoms. Our results are also consistent with previous findings of significant effects of parenting on self-esteem [38], peer attachment [40] and childhood depression [44].

There could be different explanations for the relatively small variance of parenting practices in explaining child development and the absence of direct effects of depressive symptoms in adolescence. Secure attachment in early life alone may exert only a small protective effect, in particular, if the child experiences adversity later in life [4]. Also, the experience of good parenting practices has less impact than the experience of negative parenting [13, 44], thus as individuals age, there may be no direct impact of positive parenting on depressive symptoms. Alternatively, the small effect size and the absence of a direct effect on depressive symptoms in adolescence may be due to methodological issues. We only had one measure of parenting from one parent; children may experience alternative parenting styles with different parents [60], and parenting practice may vary as the demands of child development may pose different challenges at different periods of development [62]. Given the benefits of facilitating positive parenting on child development [57], parenting practices in adolescence may also be important. Thus, future studies should use repeated measures of parenting (both negative and positive) in childhood and adolescence with a shorter time interval to explore the effect of parenting on child development.

Our finding that the developmental pathway of depressive symptoms in middle adolescence via self-esteem in late childhood and self-concept in early adolescence supports the vulnerability model (H1; [61]. Self-esteem also had a direct impact on depressive symptoms in middle adolescence. Our finding of the indirect effect of depressive symptoms in late childhood on depressive symptoms in middle adolescence via self-concept in early adolescence supports the scar model. Unfortunately, we were not able to further explore whether the vulnerability or scar effect is stronger due to the different assessments of self at different developmental stages: self-esteem in late childhood and self-concept in early adolescence. In brief, our findings suggested that across childhood and adolescence, when controlling the influence of interpersonal relationships, the association between self-evaluation and depressive symptoms may be reciprocal and should be explored further in other datasets.

Our findings of the pathway to depressive symptoms via peer difficulties alone and the pathway via friendships in late childhood and peer difficulties in early adolescence (H2) overall support the interpersonal risk model [32]. In contrast, our findings did not support the symptoms-driven model as depressive symptoms did not consistently predict peer difficulties or friendships across childhood to adolescence (H6). Surprisingly, the effect of friendships on depressive symptoms was contrary to theories and some literature [55, 58, 68] as *poor* friendships predicted fewer depressive symptoms. However, others have reported similar findings in longitudinal studies; for instance, two studies of adolescents with suicidal thoughts found a similar longitudinal association as in the current study [30, 47]. These studies suggest that friendships may not always play a protective role in relation to depressive symptoms. With a high level of intimacy, young people’s depressive symptoms might influence their friends’ affect [59],for instance, if adolescents talk about problems excessively. Besides, friendship networks and friend selection may be helpful to understand the associations detected in our study. Firstly, children with depressive symptoms are more likely to be socially excluded [10] while environmental changes such as the transition to secondary school may lead to new friendships. Then based on empirical studies from friend selection, children tend to make friends with similar characteristics [23]. Altogether, children with higher levels of depressive symptoms may move into a friendship network where individuals share a higher level of depressive symptoms.

We found some evidence to support a bi-directional association between self-evaluation and peer relationships, as previously reported [26–28, 38]. Specifically, self-evaluation predicted friendships and peer difficulties from late childhood to middle adolescence, and peer difficulties in late childhood were negatively associated with self-concept in adolescence. Our analysis was limited because ALSPAC used different self-evaluation measures in late childhood and early adolescence. More detailed exploration requires longitudinal research with repeated measures of both concepts and depressive symptoms.

We found an additional pathway from parenting to depressive symptoms in middle adolescence via peer difficulties in late childhood and self-concept in early adolescence (supporting H4). This pathway could be considered as the social-developmental origin of negative self-evaluation of depressive symptoms in adolescence [7], which is consistent with previous research [15]. This may suggest that childhood interpersonal environments play a critical role in self-evaluation because children are more likely to evaluate themselves via others’ feedback, such as peers [28].

Our analysis benefits from a robust population-based cohort study, including data from four key developmental stages, and SEM, but inevitably there are limitations. First, the assessment of child-reported friendships suffered from low internal consistency, and the evidence of the measurement invariance was weak. Although we followed previous suggestions to include mother-reported peer difficulties to assess peer relationships and applied SEM to address measurement problems for friendships [16], future studies should seek more reliable measurements to explore the role of peer relationships in depressive symptoms and our findings should be considered exploratory. Multiple informants for the same construct of peer relationships could be used to increase the accuracy of estimates for these and other variables in future studies [36]. Also, the assessment of parenting was based only on a total score, and we lacked access to individual items to assess its psychometric properties [46].

Second, the assessment of depressive symptoms in ALSPAC uses the short Mood and Feelings Questionnaire (SMFQ), which is a validated scale but not a clinical diagnostic tool. Thus, our findings cannot be generalised in clinical groups. Additionally, like many longitudinal studies, ALSPAC suffered considerable attrition which could influence the estimates of associations between variables. However, analysis of teacher-rated behaviour scores among children in or who had dropped out revealed that while the incidence was unreliable, the association between baseline predictors and teacher reported behavioural difficulties was similar in both groups [67]. This confirmed the validity of the regression model in ALSPAC samples. Despite the use of FIML as a robust and recommended approach to manage missing data [18], others have highlighted the high attrition in follow up responses to the SMFQ in ALSPAC, and multiple imputation using sociodemographic information was suggested as mitigation [35], however, this was not possible in the current study as we lacked access to such data.

Third, the current analysis could not separate between- and within- person differences and ignored trait-like individual differences in the development [25], which means that the current analysis assumed that all adolescents experience the same changes of depressive symptoms and peer relationships across time. New data analysis methods, random intercept cross-lagged panel models (RI-CLPM [25]), were proposed to address the limitation. However, we could not get RI-CLPM converged and the effect of non-invariance within RI-CLPM has not been studied yet as we failed in establishing the measurement invariance of friendships. Thus, we did not use RI-CLPM.

Due to the complexity of the proposed pathways and without specific hypotheses related to covariates, we followed Carlson and Wu [9] and have not controlled for covariates. Future studies could examine potential individual differences in the developmental pathways in different groups of children and young people. For example, family socioeconomic status influences parenting practice; Thus, it is important to include such covariates in the future pathway model. Besides, although the study provides evidence for developmental pathways of depressive symptoms, we cannot be certain that these developmental pathways are generalisable for children with neurodiversity and those with developmental disorders.

## Conclusion

Our findings highlight the importance of self-evaluation and social relationships in the relation to young people’s depressive symptoms and support the social-origin developmental pathway [7, 15]. The findings also suggest that promoting good parenting practices and healthy peer relationships in childhood may prevent the development of depressive symptoms but also promote a positive and healthy self-evaluation in adolescence. Future prevention programmes for depression in young people could be designed based on different developmental needs. While parenting programmes for parents and peer support programmes for children might benefit children, a focus on healthy self-evaluation (e.g., enhancing self-esteem, cultivating self-compassion) could be critical in adolescence.

## Supplementary Information

Below is the link to the electronic supplementary material.

Supplementary file1 (DOCX 79 KB)

## Data Availability

The data is from ALSPAC team and it is not open access. For data availability statement we can put a sentence like this: Access to ALSPAC data is through the link: http://www.bristol.ac.uk/alspac/researchers/access/.
