# Supplementary material for: Developmental pathways of depressive symptoms via parenting, self-evaluation and peer relationships in young people from 3 to 17 years old: evidence from ALSPAC
Source: Soc Psychiatry Psychiatr Epidemiol. 2023 Jan 28;58(6):907–17. doi: 10.1007/s00127-022-02416-6 (PMC10241697; doi:10.1007/s00127-022-02416-6)
Supplement: Supplementary file 1 — Supplementary file1 (DOCX 79 KB) [file 127_2022_2416_MOESM1_ESM.docx]

### Supplementary Materials

### CFA Results of Measurements

**Construct of self-esteem.** For self-esteem, the model fit of the factor structure using MLR estimator is acceptable (*χ^2^* = 313.61, *df* = 14, *p* <.001, CFI = .95, TLI = .92, RMSEA [90% CI] = .05[.05, .06], SRMR = .03). The standardised factor loadings were all significant (Table S1).

**Table S1** Factor loadings for self-esteem (standardised)

|  | Item1 | Item2 | Item3 | Item4 | Item5 | Item6 | Item7 |
| --- | --- | --- | --- | --- | --- | --- | --- |
| Factor loading | .43 | .55 | .68 | .39 | .55 | .73 | .62 |

**Construct of self-concept.** Self-concept was assessed using the Self-Image Profile (Butler, 2001). Participants were requested to use a 5-point Likert scale (from 1 always to 5 never) to rate a list of words to describe themselves. The words were listed below: *kind, friendly, funny, helpful, hardworking, talkative, confident, sporty, intelligent, be fun to be with, good looking, lazy, annoying, moody, shy, cheeky, loud, sarcastic/bitchy, bossy, short tempered, easily bored, different from others, mess about, and worry a lot.*

We tried to map the items from the Self-Image Profile (Butler, 2001) on a list of self-compassionate/positive and self-critical /negative self-referential words (Kirschner, 2016) to tap into positive self-evaluation as closely as possible. The word list of the self-referential task was developed by Kirschner (2016), and it contained 38 positive affiliative and 38 negative affiliative words. The words were listed below (alphabetical order): *adorable, afraid, alert, alone, angry, balanced, bright, calm, capable, carefree, controlling, creative, curious, depressed, detached, discouraged, distressed, docile, easy-going, embarrassed, excluded, friendly, frustrated, gentle, grateful, happy, healthy, helpless, honest, hopeful, hostile, imaginative, inferior, insecure, joyful, kind, lively, lonely, loved, loyal, lucky, mindful, moody, nervous, peaceful, protected, proud, rejected, respectful, rigid, rude, sad, satisfied, scornful, secure, self-critical, selfish, stupid, supported, suspicious, tender, tense, thoughtful, tranquil, ugly, uncertain, uneasy, unfortunate, unhappy, unpopular, unsupported, upset, useless, warm-hearted, wise, worried.*

As for selecting items, the first author (MZ) chose potential items from the Self-Image Profile (Butler, 2001) based on the self-referential task (Kirschner, 2016). Then, co-authors (AK and TF) were invited to check if the items from the Self-Image Profile (Butler, 2001) matched the meaning of the adjective word list (Kirschner, 2016). Lastly, the first author (MZ) used two thesaurus websites to double-check the match ([https://www.thesaurus.com/](about:blank) and [https://www.merriam-webster.com/thesaurus](about:blank) ).

The pilot list (14 words) is listed below. There are 7 words that we identified as self-compassionate/positive self-referential words and 7 words that we identified as self-critical/negative self-referential words. Words in bracket are from the self-referential task (Kirschner, 2016): *Kind (kind), happy(happy), friendly(friendly), helpful (thoughtful), confident (secure), intelligent (wise), good looking (ugly), moody, shy (suspicious), cheeky(rude) , sarcastic/bitch (hostile), bossy (controlling), short-tempered (angry), worry a lot (worried)*.

After selecting the words (14 items) above, CFA was used to explore the model structure. This approach was used because there is limited empirical evidence for the psychometric properties of the Self-Image Profile (Butler, 2001). Based on the items listed above, the words can be roughly divided into positive adjectives and negative self-referential adjectives. To follow factor structure of self-concept as suggested in previous literature (Esnaola et al., 2018) as closely as possible, I hypothesised a bifactor factor structure with one general factor representing self-concept and with two specific factors, positive self-referential adjectives and negative self-referential adjectives (Figure S1).

The model fit of the factor structure with all 14 words was not acceptable (Table S2), and its standardised factor loadings were listed in Table S3. We decided to omit problematic items (e.g., factor loading is not significant) to improve the overall model fit of the factor structure. Based on the Model 1, for the general factor, the factor loading of “cheeky” was not significant. Although “confident” was not significantly loaded on the specific factor either, I only used the general factor in the final structural equation model, and we only omitted one problematic word per step. Thus, cheeky was omitted, and I checked the model fit of the Model 2 with the rest of 13 words. However, the model fit of model was still unacceptable. Again, I repeated the step to omit the items, and until Model 6, the model fit was acceptable (Table S3 for factor loadings). The final words included were kind, happy, friendly, helpful, good-looking, moody, sarcastic, bossy and short-temptered.


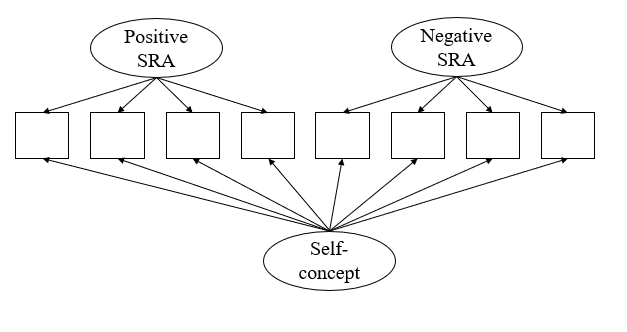


**Figure S1** *Bifactor Model Diagram of Self-Concept*

Note. Positive SRA= self-compassionate/positive self-referential adjectives; Negative SRA= self-critical/negative self-referential adjectives

**Table S2** Model fit of self-concept bifactor model

| Model |  | | MLR | | | | |
| --- | --- | --- | --- | --- | --- | --- | --- |
|  | χ^2^ | df | | CFI | TLI | RMSEA[90% CI] | SRMR |
| Model1 | 5451.30^***^ | 63 | | .86 | .80 | .111[.109, .114] | .06 |
| Model2 | 4942.35^***^ | 52 | | .86 | .79 | .112[.114, .120] | .06 |
| Model3 | 2352.21*** | 42 | | .93 | .88 | .089[.086, .092] | .04 |
| Model4 | 1784.24*** | 33 | | .94 | .90 | .088[.084, .091] | .04 |
| Model5 | 1393.14*** | 25 | | .95 | .91 | .090[.085, .093] | .04 |
| Model6 | 486.12*** | 18 | | .98 | .96 | .060[.057, .066] | .02 |

*Note.* Model1. 14 items; Model 2. 13 items; Model 3. 12 items; Model 4. 11 items; Model5, 10 items; Model 6, 9 items.

**Table S3** Standardised Factor loading of self-concept with different models

|  |  | Kind | Happy | friendly | helpful | confident | intelligent | Good-looking | moody | shy | cheeky | Sarcastic | Bossy | Short-temptered | worry |
| --- | --- | --- | --- | --- | --- | --- | --- | --- | --- | --- | --- | --- | --- | --- | --- |
| Model1 | GF | .33 | .57 | .40 | .25 | .74 | .35 | .48 | .44 | .57 | ***-.004*** | .24 | .16 | .36 | .50 |
|  | SF_PS | .80 | .41 | .71 | .57 | ***.003*** | .21 | .08 |  |  |  |  |  |  |  |
|  | SF_NS |  |  |  |  |  |  |  | .46 | -.15 | .56 | .65 | .67 | .60 | .13 |
| Model2 | GF | .33 | .57 | .40 | .24 | .73 | .35 | .48 | .44 | .58 | - | .26 | .16 | .36 | .50 |
|  | SF_PS | .80 | .41 | .71 | .57 | ***.01*** | .21 | .08 |  |  |  |  |  |  |  |
|  | SF_NS |  |  |  |  |  |  |  | .47 | -.18 | - | .59 | .70 | .62 | .13 |
| Model3 | GF | .33 | .45 | .35 | .21 | - | .22 | .28 | .66 | .37 | - | .52 | .50 | .65 | .56 |
|  | SF_PS | .77 | .50 | .74 | .58 | - | .31 | .23 |  |  |  |  |  |  |  |
|  | SF_NS |  |  |  |  |  |  |  | .12 | -.48 | - | .39 | .45 | .34 | -.29 |
| Model4 | GF | .65 | .74 | .68 | .48 | - | .40 | .46 | .40 | - | - | .30 | .20 | .38 | .28 |
|  | SF_PS | .62 | ***.06*** | .41 | .37 | - | ***-.02*** | -.17 |  |  |  |  |  |  |  |
|  | SF_NS |  |  |  |  |  |  |  | .49 | - | - | .56 | .68 | .61 | .25 |
| Model5 | GF | .42 | .41 | .39 | .28 | - | - | .21 | .21 | - | - | .50 | .48 | .67 | .37 |
|  | SF_PS | .72 | .49 | .73 | .55 | - | - | .28 |  |  |  |  |  |  |  |
|  | SF_NS |  |  |  |  |  |  |  | .58 | - | - | .55 | .45 | .26 | ***.004*** |
| Model6 | GF | .81 | .82 | .77 | .55 | - | - | .32 | .35 | - | - | .28 | .19 | .35 | - |
|  | SF_PS | .44 | -.40 | .16 | .27 | - | - | -.10 |  |  |  |  |  |  |  |
|  | SF_NS |  |  |  |  |  |  |  | .50 | - | - | .58 | .68 | .63 | - |

*Note*. Number in bold means not significant; GF = general factor; SF_PS = specific factor, positive self-referential adjectives; SF_NS=specific factor, negative self-referential adjectives;

**Construct of friendships.** The Friendships construct was assessed by five questions from the Cambridge Hormones and Moods Project Friendship questionnaire (Goodyer et al, 1989). The model fit of the one-factor structure with five observed variables (items from friendship questionnaire) was not acceptable (Table S4). Thus, we decided to omit potential problematic items and not to follow the original suggestion that an overall friendships score was computed by five questions (listed below) and response choices based on ALSPAC manual:

*F_1: “Are you happy with the number of friends you have?”*

*T2 choices: very happy, quite happy, quite unhappy, unhappy, no friends, and don’t know;*

*T3 choices: very happy, quite happy, quite unhappy, unhappy, and no friends;*

*T4 choices: very happy, quite happy, quite unhappy, unhappy, no friends, and don’t know.*

*F_2: “How often do you see you friends outside of school?”*

*T2 choices: Almost everyday, more than 1/week, once per week, less than 1/week, hardly ever, nerve, and don’t know;*

*T3 choices: Almost everyday, more than 1/week, once per week, less than 1/week, hardly ever, and nerve;*

*T4 choices: Almost everyday, more than 1/week, once per week, less than 1/week, hardly ever, nerve, and don’t know.*

*F_3: “Do you think that your friends understand you?”*

*T2 choices: Most of the time, sometimes, not often, not at all, and don’t know;*

*T3 choices: Most of the time, sometimes, not often, and not at all;*

*T4 choices: Most of the time, sometimes, not often, not at all, and don’t know.*

*F_4: “Do you talk about your problems with your friends?”*

*T2 choices: Most of the time, sometimes, not often, not at all, and don’t know.*

*T3 choices: Most of the time, sometimes, not often, and not at all;*

*T4 choices: Most of the time, sometimes, not often, not at all, and don’t know.*

*F_5: “Overall, how happy are you with your friends?”*

*T2 choices: very happy, quite happy, quite unhappy, unhappy, no friends, and don’t know;*

*T3 choices: very happy, quite happy, quite unhappy, unhappy, and no friends;*

*T4 choices: very happy, quite happy, quite unhappy, unhappy, no friends, and don’t know.*

Using SPSS, we checked the internal consistency of these 5 items. We found the internal consistency of these five questions was quite low (Cronbach's Alpha = .37(T2)/ .42(T3) /.74(T4)). In particular, the item “see friends outside of school” was not reliable as the analysis indicated that if the item omitted, the α would increase considerably, although the α was still not ideal (Table S5). We think maybe when adolescents were young, they cannot really decide if they can see their friends outside school, thus, this item does not reliably to contribute to good friendships. Additionally, based on the results of CFA (Table S4), we decided to omit the item “Are you happy with the number of friends you have” based on both theoretical and statistical reasons. From a theoretical perspective, there are three indicators of friendships, the number of friends, positive qualities of friendships and negative qualities of friendships (Schwartz-Mette et al., 2020), thus the number of friends may be not theoretically in the same latent variable with the other three questions which indicate positive qualities of friendships. From the statistical perspective, after the item was omitted, the model fits of CFA were considerably improved and were acceptable. Although the internal consistency of the three items was not high, the model fits of the CFA were acceptable at late childhood, early adolescence, and late adolescent and across three times (Table S4 for model fit, Table S6 for factor lodgings). I decided to continue using a latent variable approach in the SEM rather than summing the score as it has been shown that this can address the issue of low Cronbach's Alpha (Cole & Preacher, 2014).

**Construct of peer difficulties.** Model fits of the construct of peer difficulties at each time points could be seen in the Table S7, and the model fits were acceptable. Standardised factor loadings can be seen in the Table S8.

**Construct of depressive symptoms.** The acceptable model fits of the construct of depressive symptoms at each time points could be seen in the table S9. Standardised factor loadings can be seen in the Table S10.

### Measurement Invariance

After establishing the measurement constructs, longitudinal CFA was conducted to explore the measurement invariance (MI) of the measurements. There are different levels of MI, such as configural invariance (unconstrained factor loadings and intercepts), metric invariance (only constrained factor loadings), and scalar invariance (constrained factor loadings and intercepts). In the current study, we tried to establish the scalar invariance. Due to the big sample size, we did not use the Chi-square difference test to interpret the measurement invariance because it is too sensitive to be significant when using big sample, and we used the change of CFI (<.01) and RMSEA (<.01) as cut-off points (Putnick & Bornstein, 2016).

**Table S4** Model fit parameters of the latent variable *friendships* using MLR

| Model | MLR | | | | | | |  |
| --- | --- | --- | --- | --- | --- | --- | --- | --- |
|  |  | χ^2^ | df | CFI | TLI | RMSEA[90%CI] C.I.C.I.] | SRMR | |
| Model 1 (5 items) |  |  |  |  |  |  |  | |
| T2 (n=7444) |  | 491.84^***^ | 5 | .76 | .53 | .11[.11, .12] | .05 | |
| T3(n=6109) |  | 160.05^***^ | 5 | .93 | .86 | .07[.06, .08] | .04 | |
| T4(n=4096) |  | 554.40^***^ | 5 | .86 | .73 | .16[.15, .18] | .06 | |
| Model 2 (4 items) |  |  |  |  |  |  |  | |
| T2 (n=7444) |  | 555.21^***^ | 2 | .68 | .04 | .19[.18, .21] | .05 | |
| T3(n=6109) |  | 115.21^***^ | 2 | .94 | .81 | .10[.09, .11] | .04 | |
| T4(n=4096) |  | 514.64^***^ | 2 | .83 | .50 | .25[.23, .27] | .07 | |
| Model 3 (4 items) |  |  |  |  |  |  |  | |
| T2(n=7432) |  | 13.46^**^ | 2 | .99 | .97 | .03[.02, .04] | .01 | |
| T3(n=6108) |  | 12.66^**^ | 2 | .99 | .96 | .03[.02, .05] | .01 | |
| T4(n=4094) |  | 98.71^**^ | 2 | .96 | .88 | .11[.09, .13] | .03 | |
| Model 4 (3 items) |  |  |  |  |  |  |  | |
| T2(n=7431) |  | <.001^***^ | 0 | 1.00 | 1.00 | <.001 | <.001 | |
| T3(n=6108) |  | <.001^***^ | 0 | 1.00 | 1.00 | <.001 | <.001 | |
| T4(n=4093) |  | <.001^***^ | 0 | 1.00 | 1.00 | <.001 | <.001 | |

***Note.*** CFI = comparative fit index; TLI = Tucker Lewis Index; RMSEA= root mean square error of approximation; CI = confidence interval; SRMR = root mean square residual and standardized root mean square residual. ^*^ <.05; ^**^<.01; ^***^, <.001. n=sample size. T2 indicated friendships assessed in late childhood; T3 indicated friendships assessed in early adolescence T4 indicated friendships assessed in middle adolescence.

Model 1 indicated that there are 5 items assessing number of friends, see friends, be understood by friends, talk problem, and happy with friends.

Model 2 indicated that there are 4 items assessing number of friends, be understood by friends, talk problem, and happy with friends.

Model 3 indicated that there are 4 items assessing see friends, be understood by friends, talk problem, and happy with friends.

Model 4 indicated that there are 3 items assessing be understood by friends, talk problem, and happy with friends.

**Table S5** If the item was omitted, Cronbach's Alpha of friendships

|  | Cronbach's Alpha (5 items) | F_1 | F_2 | F_3 | F_4 | F_5 |
| --- | --- | --- | --- | --- | --- | --- |
| T2 | .37 | .30 | .52 | .25 | .28 | .29 |
| T3 | .42 | .31 | .54 | .35 | .39 | .30 |
| T4 | .74 | .68 | .76 | .68 | .70 | .66 |

***Note.*** T2 indicated friendships assessed in late childhood; T3 indicated friendships assessed in early adolescence T4 indicated friendships assessed in middle adolescence.

F_1 indicated “Are you happy with the number of friends you have”; F_2 indicated “How often do you see you friends outside of school (work)”; F_3 indicated “Do you think that your friends understand you”; F_4 indicated “Do you talk about your problems with your friends”; F_5 Indicated “Do you talk about your problems with your friends”.

**Table S6** Factor loading of friendships

|  | Model 1 (5 items) | | |  | Model 2 (4 items) | | |  | Model 3 (4 items) | | |  | Model 4 (3 items) | | |
| --- | --- | --- | --- | --- | --- | --- | --- | --- | --- | --- | --- | --- | --- | --- | --- |
| MLR | T2 | T3 | T4 |  | T2 | T3 | T4 |  | T2 | T3 | T4 |  | T2 | T3 | T4 |
| F_1 | .55 | .66 | .74 |  | .55 | .65 | .75 |  | - | - | - |  | - | - | - |
| F_2 | .10 | .16 | .44 |  | - | - | - |  | .07 | .15 | .44 |  | - | - | - |
| F_3 | .42 | .36 | .61 |  | .41 | .35 | .60 |  | .65 | .53 | .76 |  | .65 | .54 | .83 |
| F_4 | .35 | .30 | .51 |  | .35 | .30 | .50 |  | .52 | .46 | .65 |  | .52 | .46 | .63 |
| F_5 | .68 | .79 | .84 |  | .68 | .81 | .84 |  | .39 | .52 | .63 |  | .39 | .50 | .59 |

***Note.*** T2 indicated friendships assessed in late childhood; T3 indicated friendships assessed in early adolescence T4 indicated friendships assessed in middle adolescence.

F_1 indicated “Are you happy with the number of friends you have”; F_2 indicated “How often do you see you friends outside of school (work)”; F_3 indicated “Do you think that your friends understand you”; F_4 indicated “Do you talk about your problems with your friends”; F_5 Indicated “Do you talk about your problems with your friends”.

Model 1 indicated that there are 5 items assessing number of friends, see friends, be understood by friends, talk problem, and happy with friends.

Model 2 indicated that there are 4 items assessing number of friends, be understood by friends, talk problem, and happy with friends.

Model 3 indicated that there are 4 items assessing see friends, be understood by friends, talk problem, and happy with friends.

Model 4 indicated that there are 3 items assessing be understood by friends, talk problem, and happy with friends.

**Table S7** Model fit parameters of peer difficulties

| Model | MLR | | | | | |  |
| --- | --- | --- | --- | --- | --- | --- | --- |
|  | χ^2^ | df | CFI | TLI | RMSEA[90%CI] C.I.C.I.] | SRMR | |
| T2(n=8104) | 140.36^***^ | 5 | .94 | .89 | .06[.05, .07] | .03 | |
| T3(n=7078) | 117.52^***^ | 5 | .95 | .91 | .06[.05, .07] | .03 | |
| T4(n=5692) | 68.87^***^ | 5 | .95 | .90 | .05[.04, .06] | .03 | |

***Note.*** CFI = comparative fit index; TLI = Tucker Lewis Index; RMSEA= root mean square error of approximation; CI = confidence interval; SRMR = root mean square residual and standardized root mean square residual. ^*^ <.05; ^**^<.01; ^***^, <.001.

**Table S8** Factor loadings of peer difficulties

|  |  |  |  | MLR |  |  |
| --- | --- | --- | --- | --- | --- | --- |
| Time point |  | Item 1 | Item 2 | Item 3 | Item 4 | Item 5 |
| T2 |  | .41 | .52 | .66 | .49 | .51 |
| T3 |  | .48 | .51 | .66 | .55 | .49 |
| T3 |  | .50 | .54 | .55 | .42 | .49 |

*Note.* All factor loadings were significant *p* < .001

**Table S9** Model fit parameters of depressive symptoms

| Model | MLR | | | | | |
| --- | --- | --- | --- | --- | --- | --- |
|  | χ^2^ | df | CFI | TLI | RMSEA[90%CI] C.I.C.I.] | SRMR |
| T2(n=7409) | 834.11^***^ | 65 | .93 | .92 | .04[.04, .04] | .03 |
| T3(n=6076) | 1262.00^***^ | 65 | .93 | .91 | .06[.05, .06] | .04 |
| T4(n=5093) | 1736.86^***^ | 65 | .92 | .90 | .07[.07, .07] | .05 |

*Note.* All factor loadings were significant *p* < .001

**Table S10** Factor loading of depressive symptoms

|  | Item 1 | Item 2 | Item 3 | Item 4 | Item 5 | Item 6 | Item 7 | Item 8 | Item 9 | Item 10 | Item 11 | Item 12 | Item 13 |
| --- | --- | --- | --- | --- | --- | --- | --- | --- | --- | --- | --- | --- | --- |
| MLR |  |  |  |  |  |  |  |  |  |  |  |  |  |
| T2 | .48 | .33 | .35 | .25 | .68 | .48 | .44 | .66 | .36 | .62 | .63 | .61 | .53 |
| T3 | .60 | .39 | .31 | .30 | .78 | .61 | .47 | .77 | .55 | .70 | .73 | .70 | .69 |
| T4 | .63 | .57 | .44 | .45 | .83 | .60 | .50 | .80 | .72 | .71 | .75 | .75 | .81 |

*Note.* All factor loadings were significant *p* < .001

**Table S11** The results of measurement invariance

| **Model** | **χ^2^** | ***df*** | **CFI** | **TLI** | **RMSEA [95%CI]** | **Model Comparison** | **ΔCFI** | **Δ RMSEA** | **Decision** |
| --- | --- | --- | --- | --- | --- | --- | --- | --- | --- |
| Model_PD_1_ | 443.74^***^ | 72 | .967 | .952 | .024[.022, .026] |  |  |  |  |
| Model_PD_2_ | 634.18^***^ | 80 | .951 | .936 | .028[.026, .030] | Model_PD_1_ vs Model_PD_2_ | -.016 | .005 | Reject |
| Model_PD_2a_ | 537.57^***^ | 79 | .959 | .946 | .025[.023, .027] | Model_PD_1_ vs Model_PD_2a_ | -.008 | .001 | Accept |
| Model_PD_3_ | 887.72^***^ | 86 | .929 | .913 | .032[.030, .034] | Model_PD_3_ vs Model_PD_2a_ | -.030 | .007 | Reject |
| Model_PD_3a_ | 711.02^***^ | 85 | .945 | .932 | .029[.027, .030] | Model_PD_3a_ vs Model_PD_2a_ | -.014 | .004 | Reject |
| Model_PD_3b_ | 630.89^***^ | 84 | .952 | .940 | .027[.025, .029] | Model_PD_3b_ vs Model_PD_2a_ | -.007 | .002 | Accept |
| Model_FQ_1_ | 24.26^***^ | 15 | .998 | .995 | .009[.000, .015] |  |  |  |  |
| Model_FQ_2_ | 238.83^***^ | 19 | .948 | .902 | .037[.033, .042] | Model_FQ_1_ vs Model_FQ_2_ | -.050 | .028 | Reject |
| Model_FQ_2a_ | 72.08^***^ | 18 | .987 | .975 | .019[.015, .024] | Model_FQ_1_ vs Model_FQ_2a_ | -.011 | .010 | Reject |
| Model_FQ_2b_ | 35.64^***^ | 17 | .996 | .991 | .012[.006, .017] | Model_FQ_1_ vs Model_FQ_2b_ | -.002 | .003 | Accept |
| Model_FQ_3_ | 54.82^***^ | 19 | .992 | .984 | .015[.011, .020] | Model_FQ_2b_ vs Model_FQ_3_ | -.006 | .006 | Accept |
| Model_D_1_ | 5134.52^***^ | 660 | .926 | .917 | .028[.027, .029] |  |  |  |  |
| Model_D_2_ | 5814.17^***^ | 684 | .915 | .908 | .029[.029, .030] | Model_D_2_ vs. Model_D_1_ | -.011 | .001 | Reject |
| Model_D_2a_ | 5526.07^***^ | 683 | .920 | .913 | .029[.028, .029] | Model_D_2_ vs. Model_D_2a_ | -.006 | .001 | Accept |
| Model_D_3_ | 6357.35^***^ | 706 | .906 | .902 | .030[.030, .031] | Model_D_3_ vs. Model_D_2a_ | -.020 | .001 | Reject |
| Model_D_3a_ | 6081.43^***^ | 705 | .911 | .906 | .030[.029, .020] | Model_D_3a_ vs. Model_D_2a_ | -.009 | .001 | Accept |

*Note.* ModelPD_x indicated peer difficulties; ModelFQ_x indicated friendships; ModelD_x indicated depressive symptoms

###

### Missing data analysis

This section presents the results related to missing data in the current study. I created a new variable of full completed cases to label whether the participant has missing data (“having missing data labelled” as 1, “no missing data” labelled as 0). There are 1292 participants among 15645 participants without any missing data in all 80 items which assessed in the current study. Then we explored if the variables in the pathway models could predict later whether participant is in the full completed case (Table S12). Based on the regression results, we can know that the missing pattern is not completely at random.

**Table S12** Regression results

| Predictors | *β* | Estimated S.E. | *p* |
| --- | --- | --- | --- |
| Mother parenting_T2 | **-.032** | .008 | <.001 |
| Self-esteem_T2 | .002 | .011 | .850 |
| Peer difficulties_T2 | **.066** | .028 | .021 |
| Friendships_T2 | .014 | .017 | .417 |
| Depressive symptoms_T2 | .018 | .011 | .102 |
| Self-concept _T3 | .028 | .019 | .137 |
| Peer difficulties_T3 | **-.091** | .046 | .048 |
| Friendships_T3 | **.048** | .018 | .009 |
| Depressive symptoms_T3 | -.017 | .013 | .212 |
| Peer difficulties_T4 | **.066** | .030 | .027 |
| Friendships_T4 | **-.029** | .013 | .030 |
| Depressive symptoms_T4 | **.032** | .011 | .004 |

*Note.* *N*=15645; Numbers in bold were significant.

**The full list of items for parenting practices**

According to the questionnaire provided by ALSPAC team, the parenting practices were 10 activities listed below: a)bath child; b) feed child; c) sing to child; d) show child pictures in boos; e) play with toys; f) cuddle child; g) physical play (e.g., clapping, rolling over); h) take child for walks; i) put child to bed; and j) other (please tick & describe).

### References

Butler, R. J. (2001). *The Self-Image Profile for Children (Sip-C) (or for Adolescents SIP-A).*London, UK: The Psychological Corporation Ltd.

Cole, D. A., & Preacher, K. J. (2014). Manifest variable path analysis: Potentially serious and misleading consequences due to uncorrected measurement error. *Psychological Methods*, *19*(2), 300-315. [https://doi.org/10.1037/a0033805](about:blank)

Esnaola, I., Elosua, P., & Freeman, J. (2018). Internal structure of academic self-concept through the Self-Description Questionnaire II-Short (SDQII-S). Learning and Individual Differences, 62, 174-179. [https://doi.org/10.1016/j.lindif.2018.02.006](about:blank)

Goodyer, I. M., Wright, C., & Altham, P. M. (1989). Recent friendships in anxious and depressed school age children. *Psychological medicine*, *19*(1), 165-174. [https://doi.org/10.1017/s0033291700011119](about:blank)

Kirschner, H. (2016). *Compassion for the Self and Well-Being: Psychological and Biological Correlates of a New Concept* [Doctoral Thesis, University of Exeter].

Putnick, D. L., & Bornstein, M. H. (2016). Measurement invariance conventions and reporting: The state of the art and future directions for psychological research. *Developmental Review*, *41*, 71-90. https://doi.org/https://doi.org/10.1016/j.dr.2016.06.004

Schwartz-Mette, R. A., Shankman, J., Dueweke, A. R., Borowski, S., & Rose, A. J. (2020). Relations of friendship experiences with depressive symptoms and loneliness in childhood and adolescence: A meta-analytic review. *Psychological Bulletin*, *146*(8), 664-700. https://doi.org/10.1037/bul0000239
